# Sudden arrhythmic death: from basic science to clinical practice

**DOI:** 10.3389/fphys.2013.00339

**Published:** 2013-11-25

**Authors:** Ian N. Sabir, Gareth D. K. Matthews, Christopher L.-H. Huang

**Affiliations:** ^1^The Rayne Institute, St. Thomas' HospitalLondon, UK; ^2^Physiological Laboratory, University of CambridgeCambridge, UK; ^3^Department of Biochemistry, University of CambridgeCambridge, UK

**Keywords:** sudden cardiac death, ventricular arrhythmia, ion channels, action potentials, conduction velocity, re-entrant substrate, Brugada Syndrome, catecholaminergic polymorphic ventricular tachycardia

Sudden cardiac death refers to unexpected death attributable to a cardiac cause occurring within 1 h of the onset of symptoms (NICE, 2006). It often results from cardiac arrhythmias and is a major worldwide cause of morbidity and mortality. Arrhythmias account for 180,000 to 250,000 deaths per year in the United States and ~70,000 deaths per year in the United Kingdom (NICE, 2006; Chugh et al., 2008). These commonly present as ventricular fibrillation often preceded by ventricular tachycardia (Turakhia and Tseng, 2007). Cardiac arrhythmias most frequently result from underlying ischaemic heart disease (Behr et al., 2003). However, ~4% may arise from ion channel abnormalities (Tung et al., 1994; Martin et al., 2012) with their own implications for management (Martin et al., 2011). In all events, cardiac arrhythmias follow disruption of the normal cell excitation and recovery sequence propagating through successive cardiac regions. A sequence of reviews and original articles in *Frontiers in Cardiac Electrophysiology* together survey genetic, biophysical, physiological and modeling studies bearing upon mechanisms of and possible translational implications for ventricular arrhythmia and sudden cardiac death, referring to other arrhythmic situations, particularly atrial fibrillation, where these throw light upon fundamental mechanisms.

Kapur and Macrae (2013) review *developmental events* regulating appearance of molecules and structures underlying normal automaticity and conduction responsible for atrial rhythm, providing a necessary background for normal ventricular activation. Jagu et al. (2013) then outline and illustrate outcomes of *genetic studies* of biochemical processes underlying relationships between genetic background and protein expression whose alterations lead to arrhythmic tendency. Ion channel expression depends upon a sequence of processes beginning with DNA *transcription* into mRNA and its regulation by promoter sites. Persistence of the resulting mRNA then depends upon its stability and the presence or absence of mRNA splicing. *Translation* from mRNA into protein is potentially regulated by microRNAs and alternative translation phenomena. Finally, *expression* of synthesized protein depends upon its assembly, post-translational modification and trafficking to its normal membrane site.

Nielsen et al. (2013) further explore uncertainties in *relationships between genetic change and their functional consequences* specifically for Brugada Syndrome (BrS). This condition is associated with hundreds of variants in 17 genes, most commonly with *SCN5A* mutations implicating cardiac voltage-gated sodium channels. However, ~70% of BrS cases cannot currently be explained genetically. Clarification of these relationships would enhance genetic risk stratification taking advantage of multi-gene Next Generation Sequencing. However, Gütter et al. (2013) throw biophysical light on uncertainties in the relationship between genetic and functional properties: voltage-clamp investigations revealed that only one out of three mutant channels associated with clinical long QT syndrome type 3 (LQT3) and three out of six mutant channels associated with BrS showed functional abnormalities in a series of N-terminal, human hNav1.5, mutations.

Nevertheless Nielsen et al. (2013) associate more severe BrS disease phenotypes with large as opposed to small reductions in *I*_Na_, whose most obvious *biophysical effect* is to reduce action potential conduction velocity, producing potentially pro-arrhythmic *re-entrant substrate*. King et al. (2013) review factors affecting cardiac conduction velocity. The underlying local circuit current flows between myocytes depend upon not only on fast Na^+^ current, but also axial resistance and cellular excitability. These could alter with impaired Na^+^ channel and gap junction function, and altered tissue geometry following fibrotic change accompanying pathophysiological processes. Such *substrate* may accompany arrhythmic situations particularly with the *triggering* events typically associated with disrupted Ca^2+^ homeostasis exemplified by the altered sarcoplasmic reticular Ca^2+^ release through RyR2-Ca^2+^ release channels in catecholaminergic polymorphic ventricular tachycardia. Zhang et al. (2013) summarize recent studies associating *RyR2* abnormalities with atrial in addition to ventricular arrhythmias. They further point out that homozygotic *RyR2-P2328S* hearts show reduced conduction velocities potentially generating arrhythmic substrate, in addition to delayed afterdepolarization and ectopic action potential firing.

*Computational studies* permit biophysical information to be compiled into theoretical reconstructions of *in vivo physiological changes* resulting from a primary loss or gain of function. Thus, carbon monoxide (CO) is produced by a number of different mammalian tissues and exerts significant cardiovascular effects. Computational studies (Trenor et al., 2013) relate known changes in CO-induced alterations in ventricular slowly-inactivating ranolazine-sensitive late Na^+^, and Ca^2+^, channel, activity, to potentially pro-arrhythmic after-depolarization-like rhythm disturbances illustrating important elements of their underlying causes. Computational studies also illuminate *in vivo physiological outcomes* even where primary experimental systems are not available. Adeniran et al. (2013) apply these to the relatively rare, but well-defined clinical short QT syndrome, associated with accelerated ventricular repolarization, arrhythmias and sudden cardiac death. Few experimental SQT1 and SQT3 models, involving altered K^+^ or Ca^2+^ channel function are currently available. Nevertheless, application of the ten Tusscher and Panfilov (TP) human ventricular single cell model allowed *in silico* exploration of the consequences of this condition for Ca^2+^ release and mechanical output. Combined with cable theory, this further yielded whole organ functional reconstructions. This provided testable predictions implicating stretch-activated current in contractile function. Finally, an original contribution by Finlay et al. (2013) then successfully apply a modeling approach based on one-dimensional cable theory to describe human restitution dynamics incorporating both conduction velocity restitution and action potential restitution, for the first time in man.

Finally, discussions of possible *clinical translational developments* begin with problems and methodologies associated with diagnosis of sudden cardiac death risk. Vyas and Lambiase (2013) evaluate currently available *screening strategies* for sudden cardiac death risk in sudden arrhythmic death syndrome families, looking forward to roles for molecular autopsy and genetic testing, and potential future applications of stem cell-based diagnostic strategies. The first of two preliminary *clinical studies* report findings relating to sphingolipid levels as novel markers for early detection of human myocardial ischaemic injury predisposing to malignant ventricular arrhythmias (Egom et al., 2013). A report in chronic kidney disease patients, in which both early repolarization and sudden cardiac death are common, nevertheless did not associate early repolarization with increased 1-year mortality or entry onto dialysis programs (Hajhosseiny et al., 2013).

This collection of articles thus overviews current knowledge bearing on experimental studies of sudden cardiac death and its associated arrhythmias, and possible translational insights concerning clinical prevention and management.
